# Investigating the risk factors of flood deaths in Iran

**Published:** 2022-01

**Authors:** Arezoo Yari, Ali Ardalan, Yadolah Zarezadeh, Abbas Rahimiforoushani, Mohsen Soufi Boubakran, Farzam Bidarpoor, Abbas Ostadtaghizadeh

**Affiliations:** ^*a*^ Social Determinants of Health Research Center, Research Institute for Health Development, Kurdistan University of Medical Sciences, Sanandaj, Iran.; ^*b*^ Department of Health in Emergencies and Disasters, School of Public Health, Tehran University of Medical Sciences, Tehran, Iran.; ^*c*^ Department of Epidemiology and Biostatistics, School of Public Health, Tehran University of Medical Sciences, Tehran, Iran.; ^*d*^ Department of Mechanical Engineering, Urmia University, Urmia, Iran.

## Abstract

**Background:**

Floods are kinds of natural disasters that directly threaten human life. To save lives and reduce the number of injured people in floods, it is crucial to determine the underlying factors of flood deaths. This study was conducted to find the causal factors which influence flood deaths in Iran.

**Methods:**

The present research was conducted in four separate phases. In the first phase, a systematic review was conducted to determine the risk factors influencing flood death based on the available documents in the globe. In the second phase, using a qualitative study with content analysis method, the underlying factors that might cause flood deaths in different groups of Iranians were identified. In the next phase, a validated tool was developed based on the psychometry method. In the last phase, through a retrospective study using the validated tool, the risk factors affecting flood deaths were identified.

**Results:**

The systematic review identified 114 risk factors which were categorized into five groups of vulnerability factors. The results of the qualitative study indicated that a large number of underlying factors lead to flood deaths including the categories of hazard-related features, cultural, economic, social, demographic, management, and physical factors. The results of regression analysis in a retrospective study showed that by increasing some risk factors, the likelihood of flood deaths decreases. While other groups of risk factors increase the risk of flood deaths.

**Conclusions:**

Based on the findings of this study, comprehensive and appropriate strategies and interventions can be implemented to reduce and eliminate the impact of flood risk mortality and ultimately to reduce flood deaths. These include planning, training, promotion of awareness and culture of prevention, promotion of risk perception, protection of vulnerable groups, flood risk assessment and flood risk reduction, observance of urban and construction safety principles, improving urban flood management by responsible organizations, and involvement of people in all stages of death-flood risk management.

**Keywords:**

Flood-Death, Risk Factors, Iran

